# GCH1-regulated miRNAs are potential targets for microglial activation in neuropathic pain

**DOI:** 10.1042/BSR20210051

**Published:** 2021-09-10

**Authors:** Shu Jia, Guowu Chen, Yanhu Liang, Xiao Liang, Chunyang Meng

**Affiliations:** 1 Clinical Research Team of Spine and Spinal Cord Diseases, Medical Research Center, Affiliated Hospital of Jining Medical University, 89 Guhuai Road, Jining, Shandong Province 272000, China; 2 Neuropathic Pain Institute for Spinal Nerve of Jining Medical University, 89 Guhuai Road, Jining, Shandong Province 272000, China; 3 Department of Spine Surgery, Affiliated Hospital of Jining Medical University, 89 Guhuai Road, Jining, Shandong Province 272000, China; 4 Department of Clinical Medical College, Jining Medical University, 45 Jianshe Road, Jining, Shandong Province 272000, China

**Keywords:** GCH1, Microglial activation, MicroRNAs, Neuropathic pain

## Abstract

Neuropathic pain (NP) is a chronic pain directly caused by injury or disease of the somatosensory nervous system. Previous studies suggest that GTP cyclohydrolase I (GCH1) may play a pivotal role in microglial activation, which has been shown to be essential for NP. However, its underlying mechanisms in microglial activation remain unclear. A wide range of microRNAs (miRNAs) have been found to be involved in microglial activation-induced NP. To identify the miRNAs regulated by GCH1 and predict their functions in the progression of microglial activation, we analyzed the miRNA expression profiles of GCH1-knockdown (KD) BV2 microglial cells. Small RNA-sequencing analysis revealed 13 differentially expressed (DE) miRNAs in GCH1-KD cells. The target genes of DE miRNAs mainly participate in PI3K-Akt signaling pathway, peroxisome and ferroptosis. The miRNA–mRNA regulatory network analysis showed that *GCH1, MAP4K5* and *YWHAB* acted as hub genes. qRT-PCR results further verified the expression levels of mmu-miR-1a-3p, mmu-miR-133a-3p, mmu-miR-7a-5p and mmu-miR-10a-5p in GCH1-KD cells, which were consistent with the sequencing data. In addition, our data indicated that overexpression of mmu-miR-133a-3p alleviated the pro-inflammatory cytokines IL-1β and IL-6 production induced by lipopolysaccharide (LPS), indicating that mmu-miR-133a-3p has a negative effect on microglial activation. Taken together, our findings suggest that many miRNAs regulated by GCH1 may be involved in microglial activation, which may provide new potential targets for GCH1 in the pathogenesis of NP.

## Introduction

Neuropathic pain (NP) is the most common form of chronic pain and a major public health problem worldwide, with an incidence of 7–10% [1,2]. NP often results from diseases (e.g. infections, cancer) and trauma (e.g. spinal cord, peripheral nerve injury). The clinical features of NP manifest as spontaneous pain, hyperalgesia, abnormal pain and paresthesia [3,4]. Current treatments for NP, including physiotherapies and pharmacological interventions, have limited efficacy [5]. Therefore, clarifying the pathogenesis of NP is essential for discovering potential targets and novel therapeutic methods.

Microglial activation has been shown to be essential in NP [6,7]. Spinal microglial activation is induced by peripheral nerve injury and contributes to central sensitization [8]. MicroRNAs (miRNAs) have been reported to play a vital role in microglial activation-induced NP. Willemen et al. found that miR-124 exerts anti-inflammatory effects by promoting microglial M2 polarization, while reducing mechanical hyperalgesia and pain behavior [9]. Shi et al. revealed that up-regulation of miR-195 resulted in microglial activation and aggravated NP after peripheral nerve injury [10].

GTP cyclohydrolase I (GCH1), a rate-limiting enzyme in the synthesis of tetrahydrobiopterin (BH4), is essential for the activity of nitric oxide synthase (NOS) [11]. After nerve injury, the transcription of GCH1 in dorsal root ganglia (DRG) is instantly activated [12]. Down-regulation of GCH1 results in decreased microglial activation and pain attenuation, suggesting that GCH1 plays a key role in microglial activation and pain sensitivity [13]. However, the mechanism of GCH1 in microglial activation is still poorly understood.

To identify the miRNAs regulated by GCH1, we conducted small RNA sequencing of GCH1-knockdown (KD) BV2 microglial cells and explored the possible biological functions of differentially expressed (DE) miRNAs by Gene Ontology (GO) and Kyoto Encyclopedia of Genes and Genomes (KEGG) pathway analyses. The expression levels of DE miRNAs were validated by quantitative real-time PCR (qRT-PCR), and the potential effect of mmu-miR-133a-3p on microglial activation was preliminarily evaluated. The present study may be conducive to further study of the molecular mechanisms of GCH1 in microglial activation and NP.

## Materials and methods

### Cell culture and treatment

BV2 microglial cells were obtained from the Cell Resource Center, Institute of Basic Medical Sciences, Chinese Academy of Medical Sciences. Cells were cultured in Dulbecco’s Modified Eagle’s Medium (HyClone, Logan, UT, U.S.A.) containing 10% fetal bovine serum (Scitecher, France) at 37°C in 5% CO_2_. For adenovirus-mediated GCH1 KD, the shRNA sequence CCAUGCAGUACUUCACCAA was inserted into the pADV1/GFP vector, and the adenovirus was provided by GenePharma company (Shanghai, China). The cells were infected with adenovirus for 24 h, followed by maintaining in fresh complete medium, and then harvested for further study after 72 h. The sequence TTCTCCGAACGTGTCACGTT was used as negative control (NC). In order to overexpress mmu-miR-133a-3p, mmu-miR-133a-3p mimics were transfected into the cells for 48 h using Lipofectamine 2000 (Thermo Fisher Scientific, Waltham, MA, U.S.A.).

### Total RNA isolation and qRT-PCR

Total RNA was extracted with TRIzol (Invitrogen Life Technologies, Carlsbad, CA, U.S.A.). RNA quality and quantity were assessed using NanoDrop2000 spectrophotometer (Thermo Fisher Scientific, Waltham, MA, U.S.A.). Reverse transcriptase (Invitrogen Life Technologies, Carlsbad, CA, U.S.A.) was used to produce the first strand of cDNA. qRT-PCR was conducted by using cDNA and SYBR mixture (CWBio, Beijing, China) on Bio-Rad CFX-96 System (Bio-Rad, Foster City, CA, U.S.A.). Primer sequences for qRT-PCR are listed in Table 1. *GAPDH* and *U6* were used as reference genes for mRNA and miRNA, respectively.

**Table 1:** Primer sequences of miRNAs and mRNAs for RT-qPCR

Gene	Forward (5′–3′)	Reverse (5′–3′)
*mmu-miR-1a-3p*	GGGGCTGGAATGTAAAGAAGT	GTGCGTGTCGTGGAGTCG
*mmu-miR-133a-3p*	GGTTTGGTCCCCTTCA	GTGCGTGTCGTGGAGTCG
*mmu-miR-204-5p*	TCCCTTCCCTTTGTCATCCT	GTGCGTGTCGTGGAGTCG
*mmu-miR-7a-5p*	GGGGGTGGAAGACTAGTGATTT	GTGCGTGTCGTGGAGTCG
*mmu-miR-10a-5p*	GGCAACCCTGTAGATCCGAA	GTGCGTGTCGTGGAGTCG
*mmu-miR-341-3p*	GGGTTCGGTCGATCGGT	GTGCGTGTCGTGGAGTCG
*U6*	CTCGCTTCGGCAGCACATATACT	ACGCTTCACGAATTTGCGTGTC
*IL-1β*	GAAATGCCACCTTTTGACAGTG	TGGATGCTCTCATCAGGACAG
*IL-6*	CCAGAAACCGCTATGAAGTTCCT	CACCAGCATCAGTCCCAAGA
*GAPDH*	GCCAAGGCTGTGGGCAAGGT	TCTCCAGGCGGCACGCAGA

### Small RNA sequencing and identification of DE miRNAs

We constructed a small RNA library using the NEBNext Complex Small RNA Library Preparation Device of Illumina (NEB, Ipswich, MA, U.S.A.). The sequencing libraries of samples were denatured with 0.1 M NaOH to produce single-stranded DNA and sequenced by an Illumina NextSeq 500 sequencer. Cutadapt was used to trim the adapters for short reads [14]. The trimmed miRNA sequences longer than 15 nucleotides were aligned with the miRBase and genome using Bowtie. miRDeep2 was used for the prediction of new miRNAs and the quantification of miRNAs [15]. Based on CPM standardized miRNAs, the DE miRNAs were calculated and screened by R software edgeR [16,17]. The DE miRNAs between the NC and GCH1-KD BV2 microglial cells were identified as: fold change (FC) > 1.5 and *P*-value <0.05.

### GO and KEGG pathway analyses

The biological functions of DE genes were analyzed from the aspects of biological process (BP), cellular component (CC) and molecular function (MF) by GO (http://geneontology.org) analysis. The significant signaling pathways of DE genes were explored through KEGG (https://www.genome.jp/kegg/) analysis. The enrichment score was characterized by −log10 (*P*-value). *P*-value <0.05 was considered indicative of statistical significance.

### Construction of the miRNA–mRNA interaction network

The miRDB and TargetScan databases were used to predict the interactions between miRNAs and mRNAs. Cytoscape software was applied to construct the miRNA–mRNA interaction network.

### ELISA

BV2 cells were transfected with NC or mmu-miR-133a-3p mimics with or without lipopolysaccharide (LPS) treatment. The levels of proinflammatory cytokines IL-1β and IL-6 in the supernatants of BV2 cells were determined by ELISA.

### Statistical analysis

All data are presented as mean ± SEM. The statistical analyses were calculated by GraphPad Prism 7 (GraphPad Software, San Diego, CA, U.S.A.). The significance of difference between two groups was compared by Student’s *t* test. Significance was established at *P*<0.05.

## Results

### Identification of DE miRNAs

**Figure 1:**
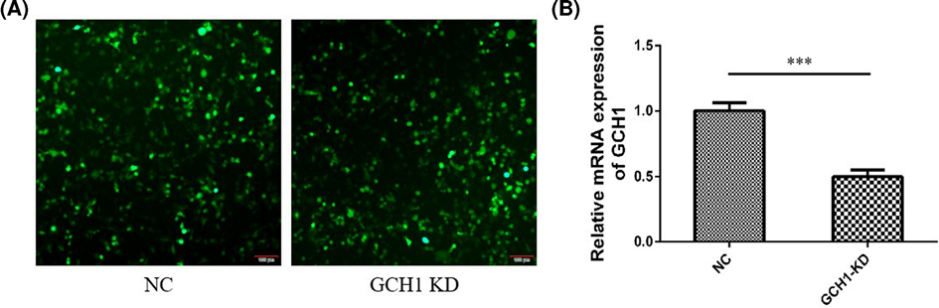
The KD efficiency of GCH1 in GCH1-KD BV2 cells (**A**) Immunofluorescence image of BV2 microglial cells infected with NC or GCH1-KD adenovirus. Scale bars, 100 μm. (**B**) The mRNA level of GCH1 in GCH1-KD BV2 cells was detected by qRT-PCR. ***P<0.001.

**Figure 2:**
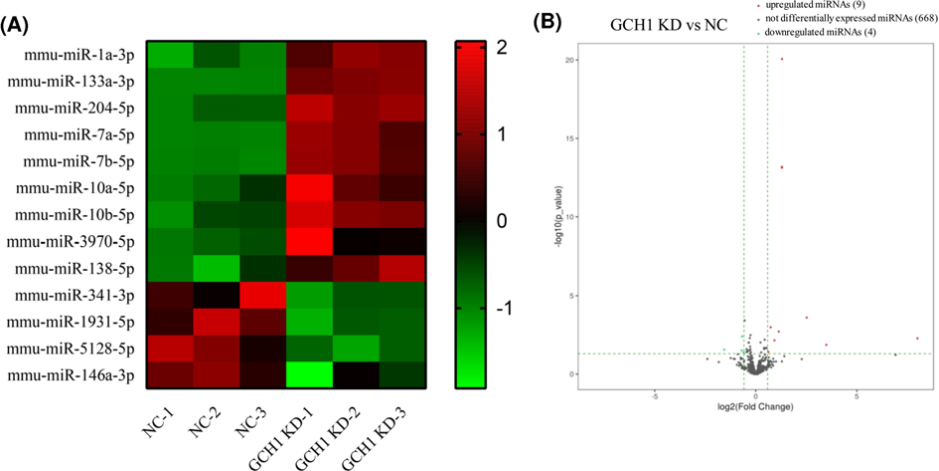
DE miRNAs between NC and GCH1-KD BV2 cells (**A**) Hierarchical clustering of DE miRNAs. Red indicates that the relative gene expression level is high, and green indicates that the relative gene expression level is low. (**B**) Volcano plots of DE miRNAs. Red dots represent up-regulated genes with >1.5-fold changes, green dots represent down-regulated genes with fold changes > 1.5, while black dots represent lack of significant differences between the two groups.

BV2 microglial cells were infected with NC or GCH1-KD adenovirus, respectively. The immunofluorescence and qRT-PCR results showed that the KD efficiency of GCH1 was close to 50% (Figure 1A,B). Small RNA-sequencing results revealed that the expression levels of 576 miRNAs were altered in the GCH1-KD BV2 cells. Using the threshold values FC > 1.5 and *P*<0.05, we identified 13 DE miRNAs, including 9 up-regulated and 4 down-regulated miRNAs. The DE miRNAs are shown in a heat map and volcano plot (Figure 2A,B).

### GO and KEGG pathway analyses

**Figure 3:**
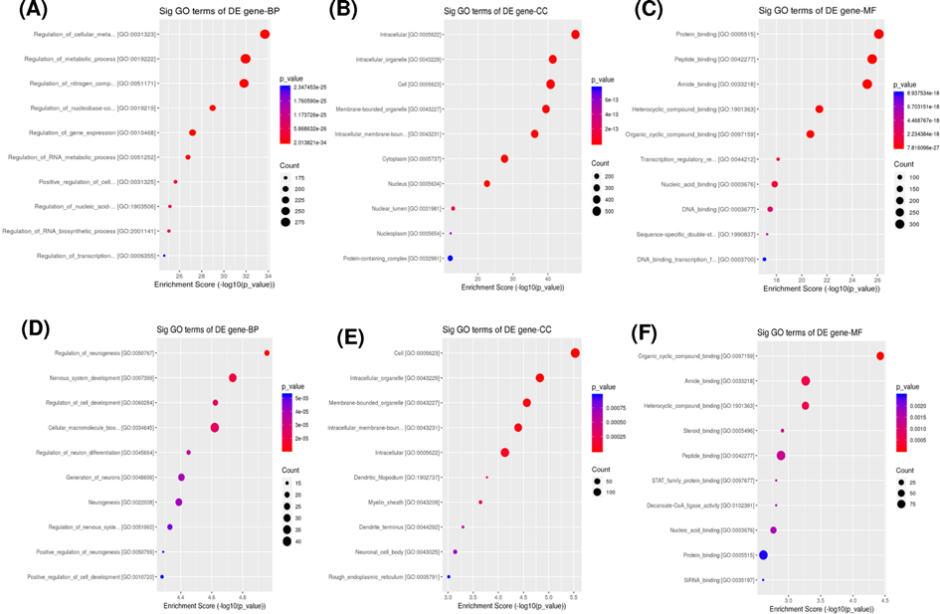
Functional enrichment analyses of the target genes of DE miRNAs using GO analysis GO analysis of up-regulated (**A–C**) and down-regulated (**D–F**) miRNAs are shown (top 10). The size of the bubbles indicates the average number of mRNAs enriched in a given pathway. The color of the bubbles indicates the *P-* value.

**Figure 4:**
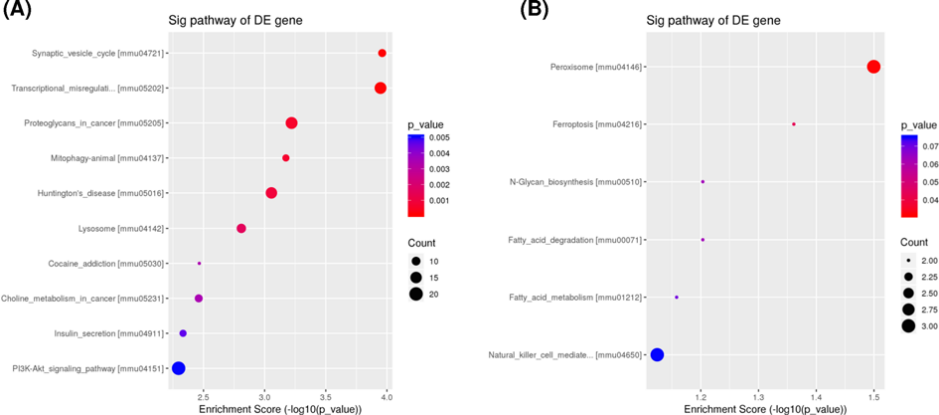
Pathway analysis of the target genes of DE miRNAs using KEGG analysis KEGG analysis of up-regulated (**A**) and down-regulated (**B**) miRNAs are shown. The size of the bubbles indicates the average number of mRNAs enriched in a given pathway. The color of the bubbles indicates the *P*-value.

The biological functions of the target genes of DE miRNAs were performed by GO and KEGG pathway analyses. The target genes of up-regulated miRNAs were mainly enriched in the BP terms related to cellular and nitrogen compound metabolic processes, while the target genes of down-regulated miRNAs were enriched in neurogenesis and nervous system development (Figure 3A,D). The most noteworthy CC terms were intracellular, membrane-bound and intracellular membrane-bound organelles (Figure 3B,E). The most enriched MF terms were protein, peptide and amide binding (Figure 3C,F). KEGG pathway analysis indicated that the target genes of up-regulated miRNAs were involved in PI3K-Akt signaling pathway and transcriptional misregulation (Figure 4A), while the target genes of down-regulated miRNAs were involved in peroxisome and ferroptosis (Figure 4B).

### Interaction network between miRNAs and mRNAs

**Figure 5:**
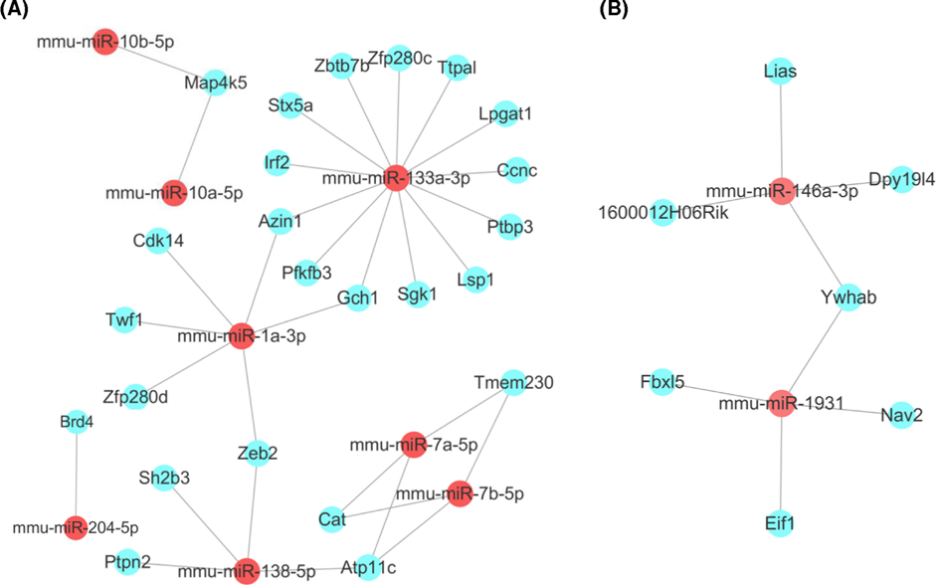
Construction and analyses of miRNA–mRNA interaction network Prediction of eight up-regulated (**A**) and two down-regulated (**B**) miRNA target genes. Red circles represent miRNAs, and blue circles represent correlated mRNAs.

The target genes of the 13 DE miRNAs in GCH1-KD BV2 cells were predicted using the miRanda and TargetScan databases. The target genes that differed between the NC and GCH1-KD BV2 microglial cells were screened using the following criteria: FC > 1.5 and *P*-value <0.05. In total, 31 target genes of the ten DE miRNAs were obtained. The target gene analysis showed that the up-regulated mmu-miR-1a-3p and mmu-miR-133a-3p both targeted GCH1, indicating that a negative feedback loop exists between GCH1 and mmu-miR-1a-3p or mmu-miR-133a-3p (Figure 5A). The up-regulated mmu-miR-10a-5p and mmu-miR-10b-5p both targeted MAP4K5. MAP4K5 belongs to the MAP kinase kinase kinase kinase (MAP4K) family, which plays an important role in a wide range of cellular responses. MAP4K5 is involved in the Wnt signaling pathway, which underlies the pathogenesis of NP [18–20]. In addition, we found that the down-regulated miRNAs mmu-miR-146a-3p and mmu-miR-1931-5p both targeted YWHAB (Figure 5B). YWHAB encodes several 14-3-3 family proteins that regulate signal transduction by binding specific Ser/Thr-phosphorylated motif-containing proteins [21]. The expression of 14-3-3ε is increased in activated microglial cells, suggesting that this protein potentially plays a role in modulating microglial activation [22].

### Verification of the candidate miRNAs by qRT-PCR

**Figure 6:**
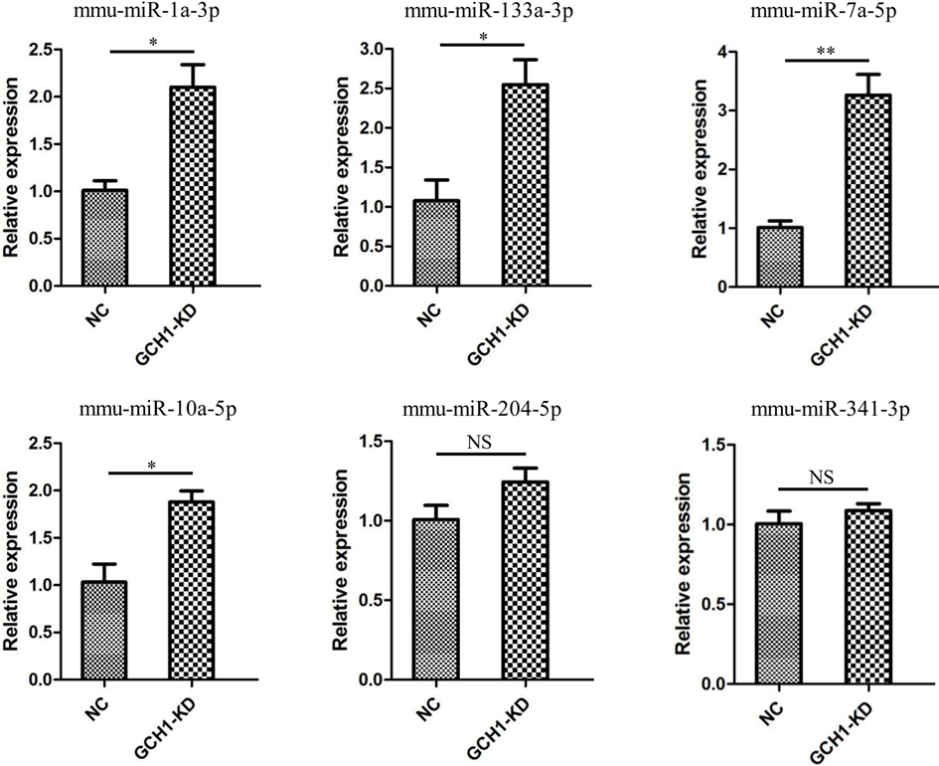
qRT-PCR of six DE miRNAs in GCH1-KD BV2 cells Data are shown as mean ± SEM. *P<0.05; **P<0.01. Abbreviation: NS, not significant.

To verify the DE miRNAs, six candidate miRNAs (mmu-miR-1a-3p, mmu-miR-10a-5p, mmu-miR-133a-3p, mmu-miR-204-5p, mmu-miR-341-3p and mmu-miR-7a-5p) were analyzed by qRT-PCR in NC and GCH1-KD BV2 cells. The results indicated that the expression levels of mmu-miR-1a-3p, mmu-miR-133a-3p, mmu-miR-7a-5p and mmu-miR-10a-5p, but not mmu-miR-204-5p and mmu-miR-341-3p, remarkably increased in GCH1-KD cells, which was consistent with the trends observed in the sequencing data (Figure 6).

### Effect of mmu-miR-133a-3p on microglial activation

**Figure 7:**
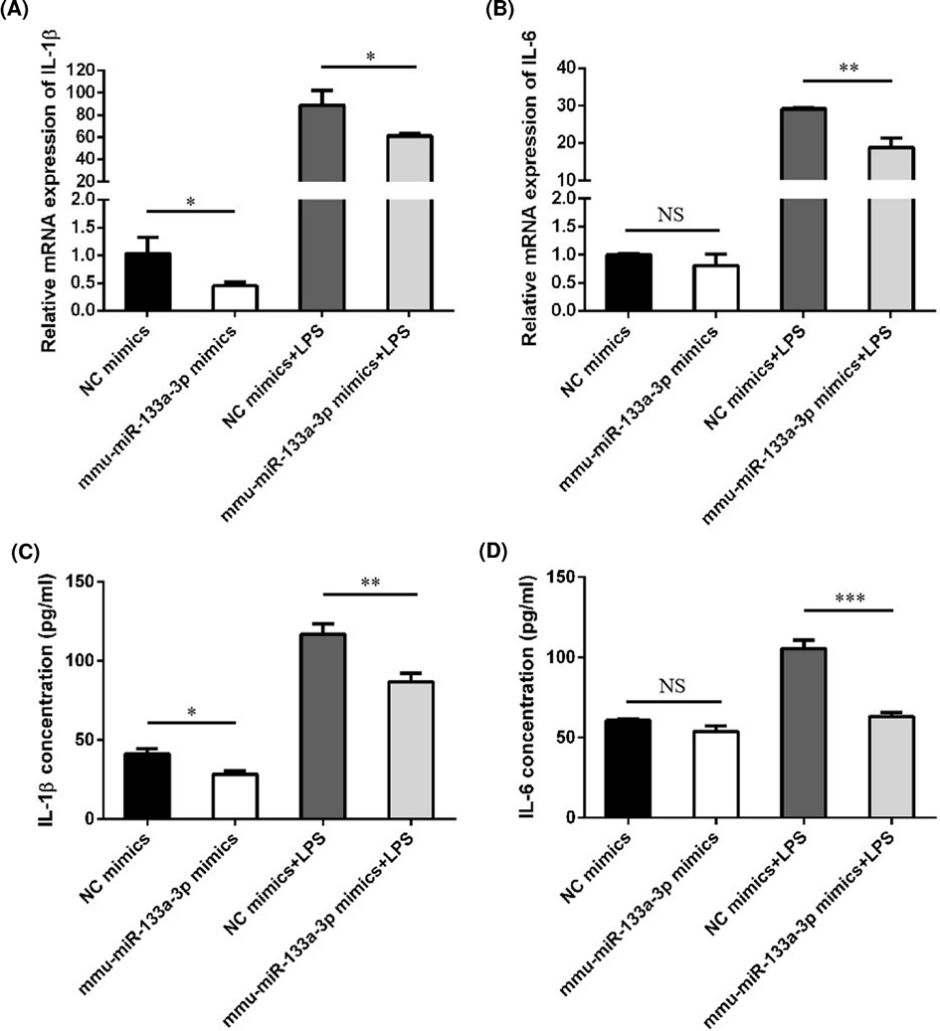
The effect of mmu-miR-133a-3p on the production of proinflammatory cytokines BV2 cells were transfected with NC or mmu-miR-133a-3p mimics followed by treatment with LPS (100 ng/ml) for 6 h. Expression of IL-1β (**A**,**C**) and IL-6 (**B**,**D**) was analyzed by qRT-PCR and ELISA. Data are shown as means ± SEM. *P<0.05; **P<0.01; ***P<0.001. Abbreviation: NS, not significant.

To confirm the roles of the DE miRNAs in microglial activation, we further analyzed the up-regulated mmu-miR-133a-3p in GCH1-KD BV2 cells. We overexpressed mmu-miR-133a-3p and examined the activation status of microglia. Overexpression of mmu-miR-133a-3p alleviated the production of the proinflammatory cytokines IL-1β and IL-6 induced by LPS, indicating that mmu-miR-133a-3p has a negative regulatory effect on microglial activation (Figure 7A–D).

## Discussion

NP seriously impairs patients’ quality of life and increases healthcare costs. The pathogenesis, pathological diagnosis and treatment of NP remain difficult. GCH1, a rate-limiting enzyme in the synthesis of BH4, was found to influence pain sensitivity. A specific ‘pain protective’ GCH1 haplotype was associated with reduced pain sensitivity in 15% of the human population [23]. Meng et al. found that the GCH1 level in nerve cells of spinal dorsal horn was elevated after peripheral nerve injury, which might contribute to the generation of NP [24]. Microglial activation has been shown to be essential for NP. Kim et al. found that GCH1 down-regulation resulted in decreased activation of microglia in the dorsal horn and pain relief, suggesting that GCH1 regulation may become a gene therapy strategy for clinical treatment of NP [13]. To reveal the underlying mechanism of GCH1 in microglial activation, we analyzed the miRNA expression profiles of GCH1-KD BV2 microglial cells by small RNA sequencing.

In the present study, we identified 13 DE miRNAs in GCH1-KD BV2 microglial cells. The target genes of DE miRNAs were mainly enriched in the following functions: cellular metabolic process, nitrogen compound metabolic process, neurogenesis and nervous system development. The pathway analysis showed that the target genes of DE miRNAs were significantly involved in PI3K-Akt signaling pathway, transcriptional regulation, peroxisome and ferroptosis.

**Figure 8:**
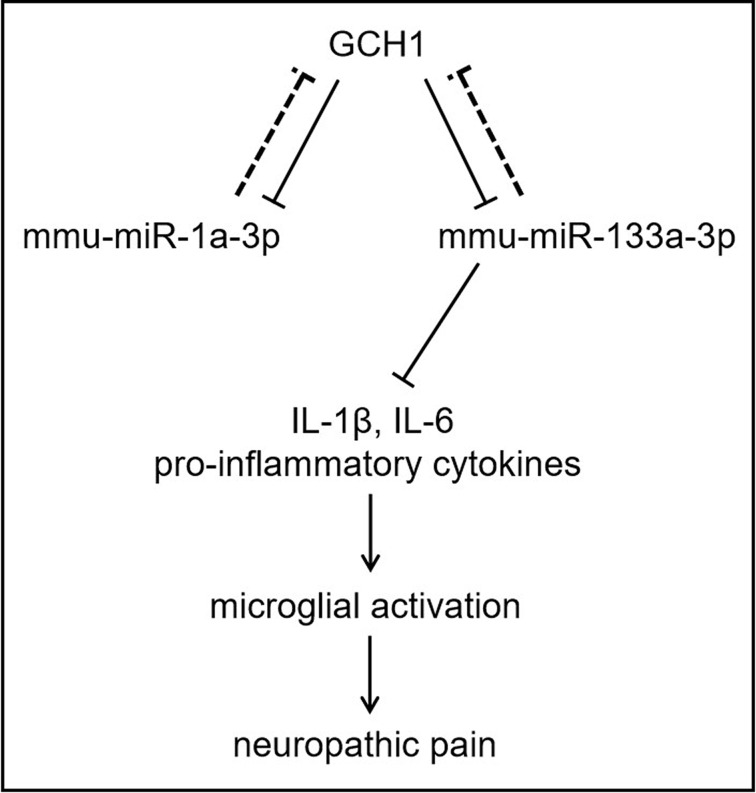
Schematic presentation of the mechanism GCH1 negatively regulates the expression of mmu-miR-1a-3p and mmu-miR-133a-3p, meanwhile GCH1 is the potential target of mmu-miR-1a-3p and mmu-miR-133a-3p. mmu-miR-133a-3p alleviated the production of the proinflammatory cytokines IL-1β and IL-6 induced by LPS, indicating that mmu-miR-133a-3p may have a negative regulatory effect on microglial activation in NP.

The miRNA–mRNA regulatory network analysis demonstrated that *GCH1*, *MAP4K5* and *YWHAB* acted as hub genes in GCH1-KD BV2 microglial cells. The target gene prediction indicated that up-regulated mmu-miR-1a-3p and mmu-miR-133a-3p both targeted GCH1. Previous studies revealed that miR-133a in the vascular endothelium regulates endothelial dysfunction by targeting GCH1 [25,26]. Although further experimental validation is needed, our results imply that a potential negative feedback loop exists between GCH1 and mmu-miR-1a-3p or mmu-miR-133a-3p. To verify the role of mmu-miR-133a-3p in microglial activation, we overexpressed mmu-miR-133a-3p and examined the activation status of microglia. The results showed that mmu-miR-133a-3p overexpression alleviated the LPS-induced production of pro-inflammatory cytokines IL-1β and IL-6, indicating that mmu-miR-133a-3p has a negative effect on microglial activation (Figure 8). Moreover, our results showed that the down-regulated miRNAs mmu-miR-146a-3p and mmu-miR-1931-5p both targeted YWHAB. YWHAB encodes several 14-3-3 family proteins, which regulate signal transduction by binding specific Ser/Thr-phosphorylated motif-containing proteins. The highly conserved 14-3-3 protein family participates in vital cellular processes, such as metabolism, apoptosis and cell cycle progression. A previous study revealed that the expression of 14-3-3ε was increased in activated microglial cells, suggesting that this protein plays a potential role in the modulation of microglial activation. These findings highlight the multiple functions and complex mechanisms of miRNAs regulated by GCH1.

Based on the fold change and *P*-value, six miRNAs (mmu-miR-1a-3p, mmu-miR-10a-5p, mmu-miR-133a-3p, mmu-miR-204-5p, mmu-miR-341-3p and mmu-miR-7a-5p) were selected to assess the expression levels. The relative expression levels of the selected miRNAs, except for mmu-miR-204-5p and mmu-miR-341-3p, were consistent with the trends observed in the sequencing data. Further experiments are needed to confirm the correlation between GCH1 and these miRNAs and the role of the DE miRNAs in microglial activation, which could provide new directions for studying NP.

## Conclusion

The present study identifies the miRNAs regulated by GCH1 and predicts their functions in the progression of microglial cell activation, which may provide new insight into the underlying mechanisms of GCH1 in the pathogenesis of NP.

## Data Availability

The datasets used and/or analyzed during the current study are available from the authors on reasonable request.

## Abbreviations

BH4: tetrahydrobiopterin
BP: biological process
CC: cellular component
GCH1: GTP cyclohydrolase I
GO: Gene Ontology
KD: knockdown
KEGG: Kyoto Encyclopedia of Genes and Genomes
LPS: lipopolysaccharide
MF: molecular function
miRNA: microRNA
NC: negative control
NP: neuropathic pain
qRT-PCR: quantitative real-time PCR
